# Climate change drives shifts in straddling fish stocks in the world’s ocean

**DOI:** 10.1126/sciadv.adq5976

**Published:** 2025-07-30

**Authors:** Juliano Palacios-Abrantes, Bianca S. Santos, Thomas L. Frölicher, Gabriel Reygondeau, Rashid Sumaila, Colette C. C. Wabnitz, William W. L. Cheung

**Affiliations:** ^1^ Institute for the Oceans and Fisheries, The University of British Columbia, Vancouver, BC, Canada.; ^2^ Emmett Interdisciplinary Program in Environment and Resources, Stanford Doerr School of Sustainability, Stanford University, Palo Alto, CA, USA.; ^3^ Climate and Environmental Physics, University of Bern, Bern, Switzerland.; ^4^ Oeschger Centre for Climate Change Research, University of Bern, Bern, Switzerland.; ^5^ Rosenstiel School of Marine and Atmospheric Science; Frost Institute for Data Science and Computing, University of Miami, Miami, FL, USA.; ^6^ School of Public Policy and Global Affairs, The University of British Columbia, Vancouver, BC, Canada.; ^7^ Stanford Center for Ocean Solutions, Stanford Doerr School of Sustainability, Stanford University, Palo Alto, CA, USA.

## Abstract

Climate-induced distribution changes are particularly challenging for fisheries targeting fish populations shared between exclusive economic zones (EEZs) and the high seas, known as straddling stocks. Here, we combine multiple datasets and ecosystem modeling to identify the presence of straddling stocks worldwide and consider the management implications of climate change–driven shifts. We identify 347 straddling stocks across 67 species, including both highly migratory and less mobile species. Our results suggest that regardless of the climate change scenario, at least 37% and 54% of stocks are projected to shift between EEZs and the high seas, by 2030 and 2050, respectively. More stocks are expected to shift toward the high seas, and highly migratory stocks are projected to shift across Regional Fisheries Management Organizations (RFMOs). Coastal states and RFMOs require to revise governance frameworks to support sustainable and equitable adaptation to shifting stocks. Developing such strategies requires cooperation between management bodies within EEZs and the high seas.

## Teaser

As straddling stocks shift between EEZs and the high seas due to climate change, fisheries will require adaptive management.

## INTRODUCTION

The biogeography of marine species is determined by historical and contemporary factors such as their evolutionary history and biology, surrounding environmental conditions, and anthropogenic factors such as exploitation. In particular, the distribution of marine ectotherms, such as fishes and invertebrates, is strongly determined by ocean conditions including temperature, oxygen, salinity, and net primary production (*1*). While fisheries management and governance are tied to human-made geographical boundaries, marine species are not. Their complex movements and habitat use can include the exclusive economic zones (EEZs) of multiple nations and waters beyond national jurisdictions (hereafter referred to as “the high seas”). Thus, many exploited fish and invertebrate “stocks” are shared across politically defined but ecologically contiguous ocean areas.

Shared stocks can be classified into four nonexclusive categories: (i) transboundary: stocks that are shared between neighboring EEZs, (ii) straddling: stocks that are shared between neighboring EEZs and the high seas, (iii) straddling highly migratory: when they straddle multiple EEZs and the high seas, and (iv) discrete high seas: when stock distribution is restricted to the high seas (*2*). Shared stocks are often managed by Regional Fisheries Management Organizations (RFMOs). Among the 18 RFMOs operating worldwide, five of them exclusively manage highly migratory species (mostly tunas): the Commission for the Conservation of Southern Bluefin Tuna (CCSBT), the Inter-American Tropical Tuna Commission (IATTC), the International Commission for the Conservation of Atlantic Tunas (ICCAT), the Indian Ocean Tuna Commission (IOTC), and the Western and Central Pacific Fisheries Commission (WCPFC). All of these RFMOs have specific areas and cover multiple species, with the exception of the CCSBT, which manages a single tuna species without a specific convention area. The other 13 RFMOs manage other straddling and discrete high-sea fish stocks such as salmon and flatfishes (*3*). Previous studies have identified thousands of transboundary stocks (*4*, *5*) with an estimated total catch of 48 million tons valued at US$77 billion in 2014 (*4*), while the catches of highly migratory species (most commercially important tuna species) were valued at US$11.7 billion in 2018 (*6*).

The management of shared stocks is more challenging than that of species restricted to a single EEZ. It depends on the proper identification of the distribution boundary, the importance of the stocks to resource users, and collaboration among resource users and managers (*7*–*9*). Thus, fish stocks are more likely to be overexploited when they are shared than when confined to a single EEZ (*10*–*12*). One of the main challenges facing the identification of shared stocks and estimation of their importance to fisheries is that stock distribution boundaries are often unclear, rendering it difficult to determine the nature of the sharing [e.g., as straddling versus transboundary (*13*)]. Accurate delineation of stock boundaries requires a robust understanding of species’ biogeography and consensus on these assessments (or evaluations) among the countries whose waters the stocks inhabit (*7*, *8*).

Climate change is adding another layer of complexity to the study, governance, and management of straddling stocks (*14*). Climate change is driving shifts in marine species distributions, with many moving toward higher latitudes, deeper waters, or along local environmental gradients, such as localized currents and upwelling systems (*15*). These distribution shifts alter the sharing of fish stocks across jurisdictional and management boundaries (*16*). Recent research highlighted consequences of such shifts for the management of transboundary stocks (*14*, *17*), including the potential for emerging transboundary stocks (*18*) and the departure of some stocks from EEZs (*19*). However, the extent of distribution shifts on straddling stocks has not been quantitatively ascertained (i.e., including the high seas), thus limiting our ability to effectively govern shared stocks under different scenarios of climate change (*20*).

This study explores the impact of climate change on the global distribution of straddling fish stocks. Specifically, we aim to identify stocks that straddle between the high seas and EEZs and investigate the effects of climate-induced distribution shifts on the proportion of straddling stocks within EEZs versus the high seas. We hypothesize that (i) straddling stocks are composed of most large pelagic species and (ii) their relative share between the high seas and the EEZ will change in the 21st century under climate change. First, we identify straddling fish stocks around the world and project their distributions using a mechanistic population dynamic model driven by outputs from three Earth system models (ESMs) under two different climate change scenarios [low-emission scenario; Shared Socio-Economic Pathway 1-Representative Concentration Pathway 2.6 (SSP1-2.6) and high-emission scenario; Shared Socio-Economic Pathway 5-Representative Concentration Pathway 5.8 (SSP5-8.5)]. Then, we examine potential climate-driven shifts in straddling stocks between EEZs and the high seas and discuss management and governance challenges for internationally shared fish stocks, highlighting the need for dynamic international strategies. Moreover, we look at changes in the distribution of highly migratory straddling stocks across tuna RFMOs. This study contributes to the growing body of literature that seeks to better understand the movements of marine species in an era of rapid environmental change, focusing on the climate-driven habitat shifts of economically important straddling stocks and implications for international fisheries management and governance.

## RESULTS

### Identification of straddling stocks

**Fig. 1.**
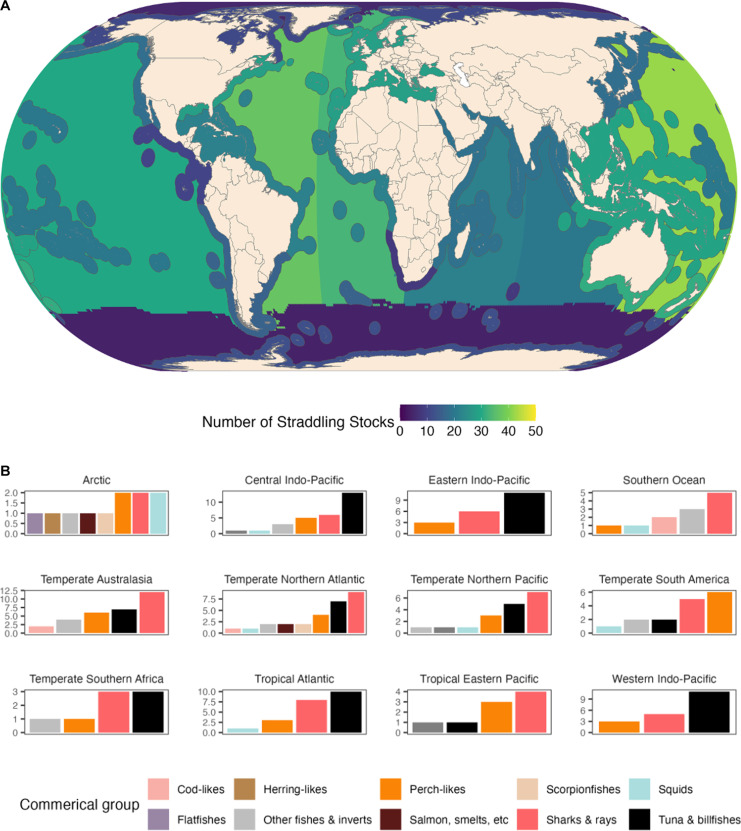
Global distribution of straddling stocks. (**A**) Map showing the number of stocks straddling between EEZs in each region and the high seas. (**B**) Number of straddling species (left axis) shared between EEZs in each region (labeled as panel titles) and the high seas with stock color coded by commercial groups according to the Sea Around Us (www.seaaroundus.org). See table S1 for species per commercial group and table S2 for EEZs per region.

Our modeling results suggest that 67 straddling commercial species [including 15 highly migratory species as defined by United Nations Convention on the Law of the Sea (UNCLOS)] comprise at least 347 straddling fish and invertebrate stocks around the world (table S1). The majority (57%) are large pelagic species. The EEZs of temperate Australasia, the central Indo-Pacific, and the temperate North Atlantic host the highest number of straddling stocks (Fig. 1A). Highly migratory species such as tunas and billfishes were the most shared commercial group in all regions. Among the top 10 most shared species were silky shark (*Carcharhinus falciformis*), blue shark (*Prionace glauca*), wahoo (*Acanthocybium solandri*), skipjack tuna (*Katsuwonus pelamis*), and yellowfin tuna (*Thunnus albacares*). The EEZs of temperate South America are the only ones not dominated by highly migratory straddling stocks. Instead, they are characterized by relatively less-mobile perch-like species such as Chilean jack mackerel (*Trachurus murphyi*), straddling the EEZs of Chile and Peru, and Patagonian toothfish (*Dissostichus eleginoides*), spanning the EEZs of Argentina, Uruguay, and the Islas Malvinas (Falkland Islands). These species are among the region’s most prevalent fish stocks.

### Shifts in straddling stocks due to climate change

We projected changes in the stock share ratio (SSR) of straddling stocks (i.e., the proportion of a stock’s abundance within EEZs versus the high seas) by 2030 and 2050 under both high and low climate change scenarios (Table 1). Our projections suggest that, considering the sum of shifts in both directions, 37 to 38% of stocks are going to have significant shifts in SSR between EEZs and the high seas as early as 2030, depending on the climate change scenario. Toward 2050, 54 to 62% of stocks show changes in SSR, under low- and high-emission climate change scenarios, respectively (Table 1). Across all scenarios and time frames, at least 37% (*n* = 123) of stocks are projected to shift (low emission scenario, SSP1-2.6 by 2030), with the majority moving out of EEZs and into the high seas (22%; *n* = 74) (Table 1).

**Table 1.** Average number and percentage of straddling stocks projected to experience significant changes in SSR across boundaries. Number of stocks represents the mean number of straddling stocks with significant changes in their SSR across ESMs in each time period and climate change scenario. Year 2030 represents the average of 2021 to 2040, and 2050 represents the average of 2041 to 2060. Minimum and maximum values per ESM represented in brackets. Direction of change indicates the direction of change of SSR. For example, “EEZ” means that the SSR is projected to increase within EEZs relative to today.

Scenario	Period	Direction of change (toward)	Number of stocks [mean (min–max)]	Percentage of stocks [mean (min–max)]
SSP1-2.6 (low emission scenario)	2030	High seas	74 (68–87)	22 (20–25)
		EEZ	49 (27–71)	15 (8–21)
		No change	220 (205–248)	64 (60–72)
	2050	High seas	116 (105–129)	34 (31–38)
		EEZ	69 (44–91)	20 (13–27)
		No change	158 (143–187)	46 (42–54)
SSP5-8.5 (high emission scenario)	2030	High seas	77 (71–85)	23 (21–25)
		EEZ	51 (28–76)	15 (8–22)
		No change	214 (197–241)	62 (57–70)
	2050	High seas	129 (125–133)	38 (37–40)
		EEZ	81 (61–103)	24 (18–30)
		No change	130 (114–153)	38 (33–44)

**Fig. 2.**
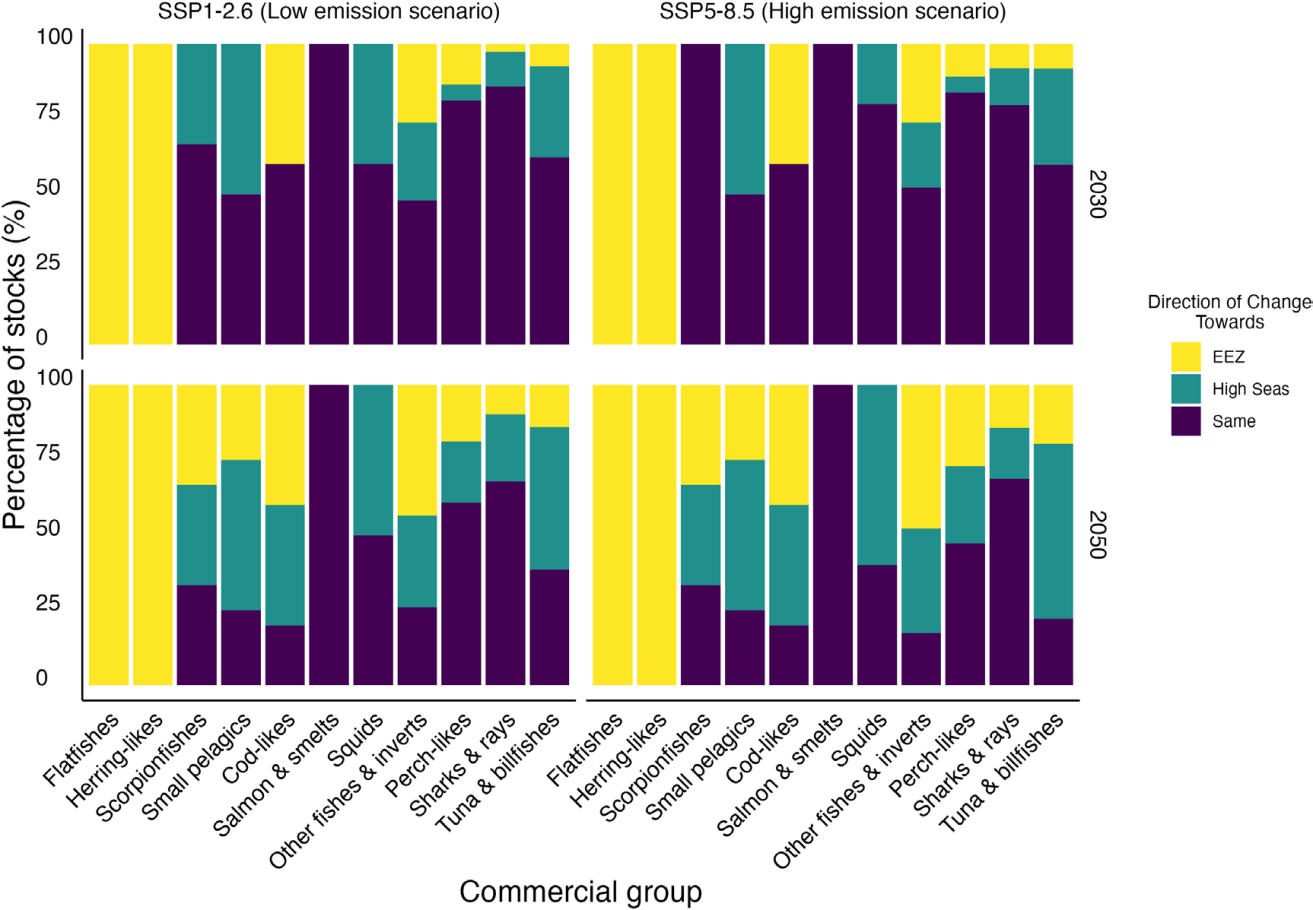
Percentage of straddling stocks shifting their distribution by commercial group. Year 2030 represents the average of 2021 to 2040, and 2050 represents the average of 2041 to 2060. Color bars indicate the direction of shift between EEZs and the high seas (yellow = toward EEZ; teal = toward high seas; and purple = no change). Commercial groups are based on the Sea Around Us (www.seaaroundus.org) classification. See table S1 for species per commercial group.

At the stock level, results show that more stocks from nearly all commercial groups are projected to have significant shifts by 2050 (Fig. 2). For some commercial groups, these shifts are going to be primarily toward the EEZs. For example, species similar to herring and flatfish, such as the American plaice (*Hippoglossoides platessoides*), which has stocks shared by Canada and the United States and is also found in the Flemish Cap area of the Northwest Atlantic Ocean high seas, are projected to shift toward EEZs. In contrast, small pelagics and squids, such as Japanese flying squid (*Todarodes pacificus*), which has stocks that straddle the EEZs of China, Japan, and Russia, are projected to move toward the high seas. Notably, only the salmon, smelts, and similar species group are projected to show no significant shift in distribution. This group includes species such as capelin (*Mallotus villosus*), which straddles the North Atlantic waters of Norway, Greenland (Denmark), Iceland, and Russia. Last, most highly migratory straddling stocks are projected to shift away from the EEZs and toward the high seas globally, especially as we approach 2050.

### Shifts in straddling stocks across international boundaries

**Fig. 3.**
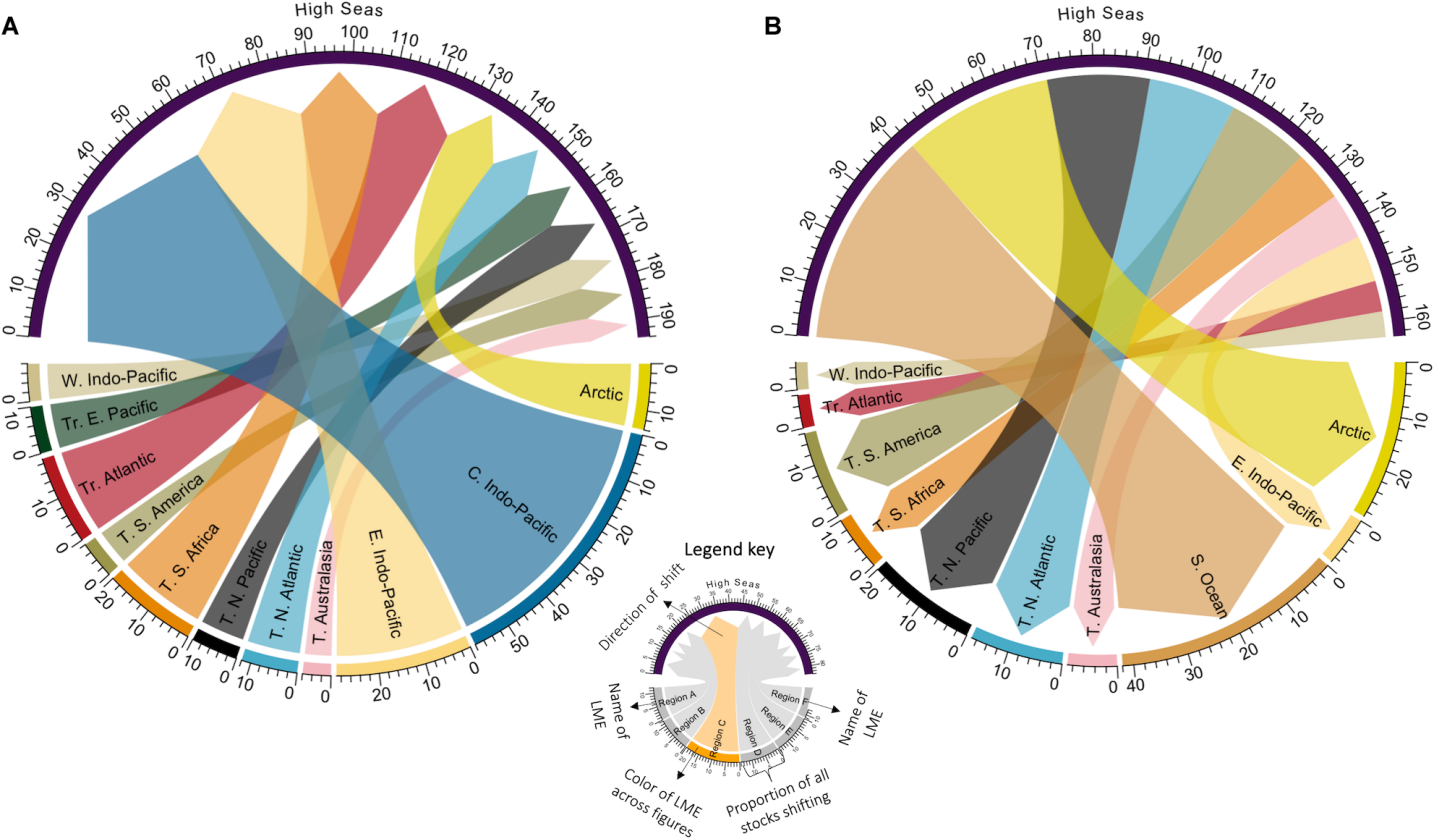
Percentage of total straddling stocks per region projected to undergo significant changes in SSR between EEZs and the high seas by 2030 under the low-emission scenario. (**A**) Arrows indicate stocks shifting from EEZs to the high seas. (**B**) Arrows indicate stocks shifting from the high seas to EEZs. Values represent the percentage of stocks experiencing significant SSR shifts relative to the total number of straddling stocks in each region. Note that the high seas total exceeds 100% because the values are aggregated across all regions. The width of each arrow link corresponds to the proportion of stocks shifting, with wider links indicating a larger proportion. For example, in (A), 12% of straddling stocks in the Arctic (yellow) are projected to shift toward the high seas. Arrows are color coded by region for clarity. C, central; E, eastern; N, north; S, southern; T, temperate; Tr, tropical; W, west; LME, large marine ecosystem. See table S2 for a list of EEZs within each region. See figure S1 for the high-emission scenario.

Our analyses show substantial regional variations in projected shift patterns of straddling stocks. Nearly all regions (*n* = 11 of 12) are projected to experience a shift of straddling stocks toward the high seas as early as 2030 (Fig. 3A and fig. S1A). Conversely, 10 regions are projected to experience stocks shifting into EEZs within the same time frame (Fig. 3B and fig. S1B). The central Indo-Pacific region (e.g., Palau, Philippines, and Solomon Islands) shows the largest variation. Here, 58% of straddling stocks are projected to increase SSR in the high seas, while no stocks are projected to increase SSR within EEZ waters (Fig. 3A). These shifting stocks include important highly migratory species such as skipjack, yellowfin, and bluefin tuna (*Thunnus obesus*), as well as non–highly migratory straddling species such as Japanese flying squid and chub mackerel (*Scomber japonicus*). A similar pattern is projected for the tropical eastern Pacific (e.g., Ecuador, Colombia, and Costa Rica), although the number of shifting stocks is smaller. As stocks move poleward under both climate change scenarios, Arctic EEZs (e.g., Greenland, Canada, and Iceland) and Southern Ocean EEZs (e.g., New Zealand and overseas territories) are projected to experience the largest gains in SSR from the high seas (Fig. 3B and figs. S1B and S2, B and D). Overall, tropical EEZs are projected to experience a net loss in SSR (calculated as the number of stocks that gained SSR minus those losing SSR) due to shifts toward the high seas, regardless of the climate change scenario or time frame considered (fig. S3). Conversely, temperate EEZs are projected to experience net gains in SSR (fig. S3A). This trend holds across all scenarios and time frames, with two exceptions: temperate EEZs in Australasia by 2050 under a low-emission scenario and South African regions by 2030 under a low-emission scenario.

**Fig. 4.**
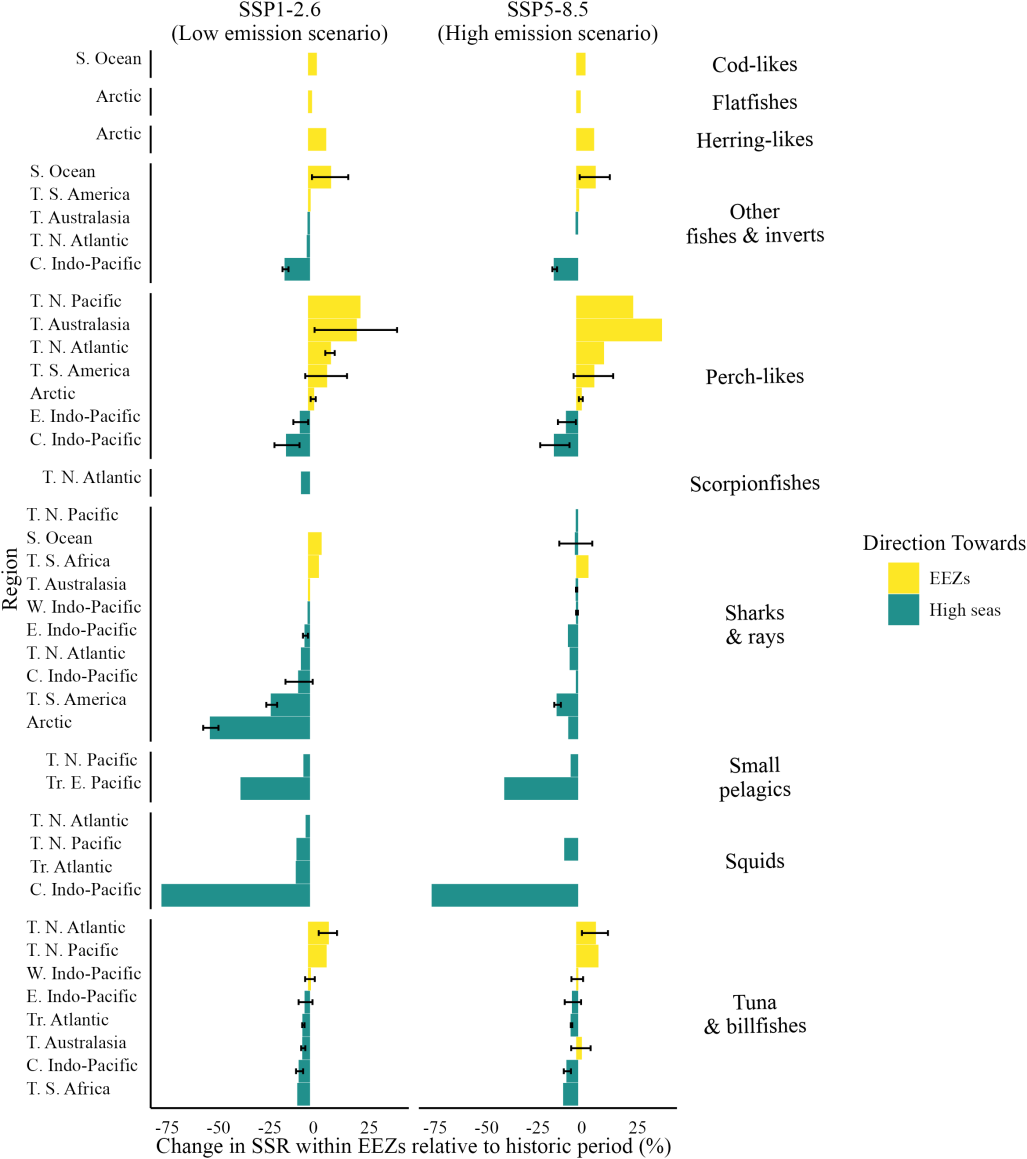
Average percentage change in SSR across commercial groups within EEZs by 2030 under both emission scenarios. Positive changes indicate an increase in the proportion of stocks within EEZs (yellow), while negative changes indicate an increase in the proportion of stocks in the high seas (green). Changes in SSR by 2030 (2021 to 2040) relative to the historical period (1951 to 2014). Error bars represent the SD of SSR changes across shifting stocks within each commercial group. Only commercial groups with significant shifting stocks are shown for each time period. C, central; E, eastern; N, north; S, southern; T, temperate; Tr, tropical; W, west. See table S2 for species per commercial group. See fig. S4 for the 2050 result.

The projected magnitude of changes in SSR between EEZs and the high seas aligns with the broader pattern of shifts (Fig. 4 and fig. S4). Most EEZs projected to see increases in SSR across commercial groups, climate scenarios, and time frames are in temperate and polar regions (Fig. 4 and fig. S4). For instance, perch-like species in temperate EEZs of Australasia are projected to increase their SSR by 50% under a low-emission scenario and up to 100% under a high-emission scenario by 2030 and 2050, respectively (Fig. 4 and fig. S4). Examples of stocks showing significant SSR changes include Atlantic mackerel (*Scomber scombrus*) in Arctic EEZs and Patagonian toothfish in temperate South America. Only two tropical regions (both in the Pacific) are expected to see slight increases (both on perch-like species): the western Indo and tropical eastern Pacific. Here, perch-like species are projected to increase SSR by 1.3 to 1.5% under a low-emission scenario by 2050 and 3% under a high-emission scenario. On the other hand, most tropical EEZs are projected to experience significant losses in SSR, regardless of the time frame or climate change scenario (Fig. 4 and fig. S4). A notable example is squid in the central Indo-Pacific, where SSR within EEZs is projected to decrease by 75% by 2030 under both climate scenarios (Fig. 4) and by 85 to 89% by 2050 under low- and high-emission scenarios, respectively (fig. S4). While polar and temperate EEZs generally show slight reductions in SSR, the Arctic stands out with a projected loss of 50% in SSR for sharks and rays under a low-emission scenario by 2030 (Fig. 4).

### Shifts in highly migratory straddling stocks across RFMOs

**Fig. 5.**
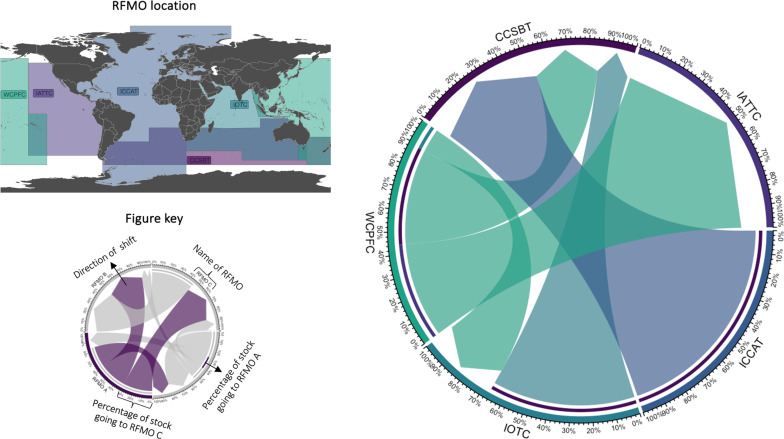
Net changes in SSR of highly migratory straddling stocks between neighboring RFMOs by 2030 under the low-emission scenario. Links colored by RFMO. Net changes = stock gains – stock losses. Net changes in SSR by 2030 (2021 to 2040) relative to the historical period (1951 to 2014). Numbers on the axis and size of links represent the percentage of stocks shifting, and the arrow shows the direction of the shift. For example, 80% of net changes from IOTC will represent stocks shifting toward CCSBT, while 20% will come from WCPFC. The Commission for the Conservation of Southern Bluefin Tuna (CCSBT), The Inter-American Tropical Tuna Commission (IATTC), The International Commission for the Conservation of Atlantic Tunas (ICCAT), The Indian Ocean Tuna Commission (IOTC), and The Western and Central Pacific Fisheries Commission (WCPFC). See fig. S5 for the high-emission scenario result.

We projected shifts of highly migratory straddling stocks (i.e., most tuna and billfishes and sharks and rays) between neighboring RFMOs in addition to shifts between RFMOs and EEZs (see Materials and Methods). We found that 19 and 25% of stocks are projected to shift across RFMOs by 2030 under the low- and high-emission scenarios, respectively (Fig. 5 and fig. S5). By 2050, the number will increase to 32 and 41% under the low- and high-emission scenarios, respectively. Overall, we projected eastward and poleward net changes in the SSR of highly migratory stocks between neighboring RFMOs (Fig. 5 and fig. S5). By 2030, IATTC and CCSBT are projected to have net gains of SSR from all neighboring RFMOs, as opposed to ICCAT and WCPFC. IOTC is the only RFMO projected to gain and lose stocks from neighboring organizations. This includes the potential shift of highly migratory stocks toward CCSBT, as well as shifts from the WCPFC area in the Java Sea where these two border (Fig. 5). This trend is the same under a high-emission scenario and by 2050 under both climate change scenarios (fig. S5).

## DISCUSSION

This study identified the extent to which EEZs around the world have stocks that straddle the high seas and the potential changes in proportion across boundaries under two climate change scenarios. Here, we discuss the implications of such shifts for the management and governance of important economic species. Our findings highlight that most straddling stocks are large pelagic fishes and that the existence of multiple non–tuna-like stocks straddling the waters of the world’s EEZs and the high seas is not represented in UNCLOS Annex 1 or lists, created using knowledge from RFMOs (*21*, *22*). However, these species are reported to be caught in the high seas [e.g., (*23*–*25*)]. Addressing taxonomic gaps in RFMO governance and management structures is critical to improving fisheries and ocean health. Most RFMOs tend to only manage a small portion of all species affected by fishing activities within their convention zones. Expanding taxonomic mandates of existing RFMOs can be useful for a more ecosystem-based approach to fisheries management (*26*). It is very likely that our analysis underestimates the number of straddling species, as our initial data only include stocks that are commercially important to global fisheries (*4*, *27*), and ocean biodiversity comprises substantially more taxa (*28*).

We project changes in the shared proportion of straddling stocks independent of the climate change scenario (low- and high-emission scenarios) in the next decades, posing challenges to the management of shared stocks. The climate change scenario independence of our results can be attributed to oceanic changes projected during the early part of the century being very similar between both climate change scenarios considered here (*29*). While maintaining global warming levels below 2°C relative to preindustrial levels will partially mitigate marine fish biomass loss (*30*) and impacts to fisheries (*31*), management actions must still prepare for potential climate-driven shifts in straddling stocks (Table 1). This means that, even if global warming is kept below 2°C, climate change will still have an effect on marine biodiversity and associated fisheries, including those species capable of fast shifts (*32*). Over the past couple of decades, climate change has gained increased attention from RFMOs. For example, ICCAT has a revised plan of action to guide the commission and the standing committee on research and statistics on relevant issues associated with climate change (*33*). At the same time, the WCPFC is in the process of adopting hard limits for the high-seas purse-seine fishery, which would provide a mechanism for ensuring that the benefits to Pacific small island developing states are respected as tunas shift eastwards toward the high seas (*34*–*36*).

Developing management approaches that respond to climate impacts in a precautionary or adaptive way can be politically difficult (*35*, *37*, *38*). Today, most regional fisheries bodies that address climate change do it in a procedural or administrative way that will need to markedly change to cope with shifting stocks (*35*). Structurally, most RFMOs have the capacity to respond constructively to climate change, as they operate under consensus-based decision-making policies that can be a powerful tool for navigating complex issues across diverse national goals. However, these structures may limit management implementations (*39*, *40*), as member states can have opposing priorities and differing interests (*41*). Moreover, there are limited accountability mechanisms in place to ensure RFMOs adequately protect straddling stocks, despite legal frameworks (e.g., UNCLOS/UN Fish Stocks Agreement) (*21*, *42*) specifically articulating expectations for states to cooperate and coordinate (*37*). From a technical perspective, incorporating climate change into ocean management plans aimed at protecting biodiversity (*43*, *44*), rebuilding overfished stocks (*45*), and governing fisheries (*46*, *47*), including those targeting transboundary stocks (*8*, *17*, *18*), will be key to effectively respond to the impacts of climate change on marine ecosystems. Guidelines can include specific rules to anticipate shifts in straddling stocks, such as considerations around the addition of nations to governance frameworks as stocks expand to regions where they were not before, compensation of party member(s) who lose stock shares (*48*), and adaptive and equitable management strategies (*49*, *50*).

The governance of straddling stocks is expected to become increasingly complex, as these stocks shift their distribution across the boundary of EEZs and the high seas in response to climate change (*20*). Shifts leading to substantial increases in biomass on the high seas could pose threats to sustainable fisheries management especially in areas without RFMOs or rules in place to effectively manage shifting stocks (*51*). These shifts also have the potential to spark international conflicts, as evidenced by disputes near the boundaries of the Argentinean and Ecuador (Galápagos) EEZ—areas where fishing vessels’ automatic identification systems (AISs) are often disabled (*52*, *53*). While the disabling of AIS does not necessarily indicate illegal fishing, squid jiggers are among the vessels most commonly associated with this practice (*53*). This is particularly concerning given that many stocks of these species are projected to increase their biomass on the high seas (Fig. 2 and 4). Notably, only a limited number of wealthy nations have the capacity to access the high seas (*54*). As a result, the shift of stocks onto the high seas is likely to disproportionately affect island and coastal nations, whose national revenue relies, in part, on access agreements with foreign flag nations to fish within their waters, for instance, resulting in substantial socioeconomic losses (*36*, *55*). In addition, these shifts could also lead to conflicts between local nations and distant water fishing nations operating near EEZ boundaries (*53*). These shifts may also exacerbate risks of food and nutrition insecurity of coastal nations that are heavily dependent on fish for their culture, livelihoods, and diets (*54*). Heterogeneities in governance capacity across EEZs further complicate governance challenges. For some states and territories, the movement of stocks from an EEZ-managed area to the high seas may result in decreased governance effectiveness or loss of revenue. Conversely, other regions may experience improved governance and increased benefits under such circumstances. To address these challenges, a more detailed assessment of domestic capacity for managing fish stocks along with the economic importance of coastal (and oceanic) fisheries across different EEZs is key. These assessments will provide deeper insights into regional governance challenges and help develop more equitable and effective management strategies for shifting stocks.

More explicit efforts will be needed to address potential future losses for coastal states as stocks shift toward the high seas, with likely major economic repercussions. Most, if not all, potential solutions necessitate improved collection of scientific data and coordination of research efforts to inform proactive management approaches and underpin robust governance structures (*17*, *56*). For example, reliable stock estimates to allow for the allocation of quotas among member states under a changing climate will require the strengthening or development of scientific research programs that include the collection of species’ life history and abundance data from all jurisdictions in which species are to be fished (*57*–*59*). However, current global data and data collecting systems (i.e., fishery-independent scientific surveys) may be insufficient and have spatial-temporal sampling biases that need to be addressed to properly manage straddling stocks in a changing climate (*60*, *61*).

There have been increasing calls to improve monitoring, control, and surveillance of fishing activities among RFMOs, such as through improved coverage of observer programs or vessel monitoring systems (*62*, *63*). Effective management of straddling stocks, including expanding taxonomic mandates, can be useful for a more ecosystem-based approach to fisheries management and requires cooperation between RFMO member states (*64*). However, these might be unrealistic without complementary approaches that assist in providing monitoring and species assessments (*26*) and enforcing compliance (*65*). As existing structures often limit their capacity to deliver timely responses, international fora such as the United Nations Biodiversity of Areas Beyond National Jurisdiction Treaty (BBNJ) may provide opportunities to raise and devise mechanisms to address these challenges. Under the BBNJ, RFMOs are expected to play a key role in the establishment of area-based management tools, providing a vehicle for RFMOs to ensure that their management and conservation objectives are considered and valued and align with broader international efforts (*66*). The BBNJ may offer a legal framework for global and regional climate action for protecting marine biodiversity in a changing climate and create a legal obligation to consider climate change in the process of environmental impact assessment of activities on the high seas (*67*). However, ensuring timely and effective responses will likely remain a substantial challenge (*16*, *37*).

The shifts in highly migratory stocks, such as tunas and billfishes, will require RFMOs to coordinate actions and align management measures among them to avoid potential conflict and resource overexploitation (*68*). While there are ongoing efforts and a recognized need for robust mechanisms to ensure sustainable fisheries management amidst climate-induced stock redistributions, inter-RFMO collaboration in response to climate change is still developing, with few specific policies addressing the uncertainties introduced with climate change (*38*). Notably, WCPFC and IATTC have been actively exploring legal and practical frameworks for better collaboration for some time, including shared governance structures (*40*, *69*, *70*). WCPFC and IATTC are already faced with overlapping management areas and stocks, a situation that is likely to intensify as tuna and tuna-like stocks shift eastward across the tropical Pacific (*6*, *36*, *71*). Similarly, for non–highly migratory straddling stocks, the Northwest Atlantic Fisheries Organization and the North East Atlantic Fisheries Commission have worked together for more than 15 years to jointly manage a pelagic oceanic redfish stock that became shared because of a climate-driven distribution shift. Furthermore, both the International Pacific Halibut Commission and the Pacific Salmon Commission in the Northwest Pacific are actively investigating the impacts of climate change on halibut and salmon stocks, respectively, successfully overcoming competitive tactics that have jeopardized the sustainability of these stocks in the past (*72*). Research endeavors and coordinated conservation and management measures similar to these could provide some important lessons learned for other RFMOs stepping into this space.

Given the challenges of a global-scale analysis of shared stocks under climate change, our findings should be cautiously interpreted given the caveats and uncertainties of the data, model assumptions, and analytical methods. First, our estimation of the number of straddling stocks is subject to the original species distribution data, the geographical borders of EEZs, their classification on marine realms (e.g., what EEZs fall into which marine regions), and the spatial thresholds selected for analysis (figs. S6 to S8). Only 9% (*n* = 7) of the species identified as straddling are not reported on Annex I of UNCLOS (*21*), identified by experts (*22*), or recorded in FishBase (www.fishbase.org) as having high-sea distributions. While these corroborations provide a certain level of confidence in our results at the species level, future studies that focus on particular straddling stocks should aim to use a more specific unit stock definition and distribution boundary. Second, our projections are based on three ESMs and one single marine ecosystem model (MEM). Using three ESMs allows us to capture some of the uncertainty inherent in climate change projections (Table 1); along these lines, incorporating an ensemble of MEMs that provide species-level data could further address projection uncertainty, leading to more reliable results (*73*, *74*). Moreover, global models are intended to show large-scale processes that might not be well suited for smaller scales, as they fail to capture local processes (*75*). Ideally, global studies should encourage local to regional efforts to apply more detailed approaches to a specific question; however, these can still be useful in cases where no other options exist (*76*), especially given the uncertainty that exists between global and regional models’ outputs (*77*). Third, future species distribution projections can be influenced by interactions between species (*78*), adaptation of species to environmental changes, and anthropogenic factors (*79*), all of which are not captured by the dynamic bioclimatic envelope model (DBEM). Previous studies coupling multiple MEMs ranging from size-based to trophic-based to species distribution to functional models have shown similar (negative) trends in changes in global marine biomass under climate change with the DBEM overall performing at the middle range of the ensemble (*30*, *80*).

Our analysis identified 67 key species, most of which are large pelagics, that encompass at least 347 straddling commercial fish stocks globally, with the highest concentrations in the temperate North Atlantic, temperate Australasia, and the central Indo-Pacific regions. Projections indicate that while the SSRs of most straddling stocks remain stable in 2030, notable shifts are observed for some important stocks. By 2050, a substantially larger number of stocks are projected to shift, with the magnitude of change being particularly pronounced under a high-emission scenario. As a result, coastal nations and RFMOs urgently need to adapt their management and governance structures to account for and accommodate these shifts and their associated impacts to ensure sustainable and equitable resource management, incorporating dynamic stock assessments, adaptive management strategies, and enhanced international cooperation.

## MATERIALS AND METHODS

### Defining straddling stocks

In this study, we define a straddling stock as one that is present in two or more neighboring EEZs and their adjacent high-sea areas. We subidentify highly migratory straddling species as those listed in the UNCLOS Annex I at the species level, which includes 12 tuna species, nine marlin species, two sailfish species, one swordfish species, four saury species, two dolphin species, and five shark species (*21*). We consider 19 of these in our analysis (see table S1). However, UNCLOS does not provide guidance on how these were classified or any guidance or identification of less-mobile straddling stocks. Because explicit criteria for identifying non–highly migratory straddling fish stocks were not available at the time of this study, we developed a set of steps to operationalize the identification of these stocks and enable subsequent quantitative analysis of straddling stock shifts. We adapted a three-criterion approach to determine whether a stock is to be considered straddling. This method was selected on the basis of its reliance on empirical information from multiple data sources and has been previously used to identify transboundary stocks (*12*, *14*). The three criteria included (i) agreement among data sources, (ii) identifying neighboring EEZs and the high seas, and (iii) determining a spatial distribution threshold. Only stocks that met all three criteria were considered straddling stocks.

1) Agreement among data sources: We used occurrence records and distribution range maps of the species from four data sources (two observational and two modeled) to determine the presence of each species within a 0.5° by 0.5° grid of the world’s oceans. To increase the certainty of species occurrences and the robustness of subsequent analyses, records that were not supported by all the data sources were not used in our analysis. The observational data sources included occurrence data collected from five publicly available repositories (*28*) and the Sea Around Us (SAU) global fisheries catch dataset from 1950 to 2014 that was available for the analysis (*27*). Because of gaps and sampling biases in the occurrence records (*61*), we supplemented them with published range maps predicted from two different species distribution approaches. The first consists of a multimodel approach based on an ensemble of species distribution models: Bioclim and boosted regression tree model (*81*), Maxent model (*82*), and nonparametric probabilistic ecological niche model (*83*). These statistical approaches are a common tool to determine the relationship between observation records and environmental variables following the ecological niche theory (*28*, *84*). The second model adopted by SAU follows a five-step process including catch reports on major fishing areas as defined by the Food and Agriculture Organization and the country’s EEZs, species-specific life history, expert review, habitat preference, and equatorial submergence (*85*, *86*). Last, species distributions from all data were limited by their depth preferences. All data were gridded at 0.5° latitude by 0.5° longitude resolution.

2) Neighboring EEZs and the high seas: A stock was considered straddling only if it was shared between two or more neighboring EEZs and the high seas. To identify such stocks, we subdivided the high seas into west-east biomes (*87*) and matched EEZs to marine realms according to the marine ecoregions of the world (*88*). All EEZs were grouped into one of these 12 realms (fig. S7 and table S2). Because phenotypic or phylogenetic delineations of discrete populations of straddling species are limited to a few well-studied species (*5*, *22*), we used realms as a proxy for stock boundary delineation. Realms represent large spatial units across which biota are internally coherent at higher taxonomic levels as a result of a shared evolutionary history (*88*). To further refine the analysis, we identified the ocean basin immediately adjacent to each realm. This approach is considered a conservative stock delineation; for example, a species such as Atlantic cod is represented as two distinct stocks visiting the high seas: one straddling the waters of North America and the western Atlantic Ocean and another straddling the waters of Northern Europe and the eastern Atlantic Ocean. For the 19 highly migratory straddling species identified in UNCLOS Annex I, we used the five RFMOs tasked with managing highly migratory fish stocks on the high seas (fig. S7): the CCSBT, IATTC, ICCAT, IOTC, and WCPFC. CCSBT is responsible for the management of southern bluefin tuna throughout its distribution without a convention area. For this analysis, the CCSBT area has been considered as the area south of IATTC, ICCAT, IOTC, and WCPFC convention areas’ southern limits (fig. S6).

3) Spatial distribution threshold: We computed the proportion of a given stock overarching the shared distribution between the neighboring EEZs and the high seas and set a minimum threshold. Here, we classified a stock as straddling if both the EEZs and the high seas enclosed at least 10% of a stock’s joint shared distribution. We examined the sensitivity of the identification of straddling stocks to the assumption of a shared stock threshold using >0, 20, and 30%. We expected that the threshold level would influence the number of straddling stocks with “no threshold,” resulting in substantially more stocks, especially non–highly migratory species (fig. S7).

### Climate change projections

We projected changes in abundance and maximum catch potential [a proxy for maximum sustainable yield (*89*–*91*)] of each straddling stock from 1951 to 2100 using a DBEM. The DBEM has been extensively described, and thus, we only provide an overall description of the model here (*89*–*91*). The DBEM projects relative abundance of a species on a 0.5° latitude by 0.5° longitude grid based on environmental variables (i.e., habitat suitability) and preferences (e.g., sea temperature, salinity, oxygen concentration, bathymetry, and, for polar species, sea ice), known depth and latitudinal ranges, and spatial population dynamics. Species-related data for the DBEM were obtained from FishBase (www.fishbase.org) and SeaLifeBase (www.sealifebase.org) for fish and invertebrate species, respectively.

Recognizing the variations in the ESM projections (*29*, *92*), the DBEM simulations were driven by outputs from three ESMs that participated in phase 6 of the Coupled Model Intercomparison Project. The ESM outputs used were annual mean bottom and surface oxygen, pH, salinity, and potential temperature. ESM output also included sea ice cover, current velocity, and total net primary productivity (NPP), NPP of picophytoplankton, and NPP of diatoms vertically integrated over the top 100 m. The three ESMs included were the Geophysical Fluid Dynamics Laboratory–ESM4 (*93*), the Institut Pierre-Simon Laplace–CM6A-LR (*94*), and the Max Planck Institute Earth System Model–ESM1.2 (*95*). Climate change projections followed two contrasting scenarios according to the SSPs and RCPs: SSP1-RCP2.6 (SSP1-2.6) and SSP5-RCP8.5 (SSP5-8.5) scenarios (*96*, *97*). The SSP1-2.6 is a low emission–high mitigation scenario with radiative forcing stabilized at 2.6 W/m^2^ by the end of the 21st century. The SSP5-8.5 scenario is a high emission–low mitigation scenario with radiative forcing of 8.5 W/m^2^ by the end of the 21st century.

### Estimating straddling shifts

We calculated the number and magnitude of shifts in straddling stocks between EEZs and the high seas. For each group of EEZs within the same realm and ocean biome (for straddling) and RFMO (for highly migratory straddling), we calculated the annual total abundance of each straddling stock from 1951 to 2100 under each ESM and climate change scenario. We then estimated the yearly SSR between each region and the high seas (*98*). The SSR quantifies the proportion of a stock’s abundance located in EEZs versus the high seas. For example, if the EEZs of the temperate Atlantic region hold 80% and the high seas 20% SSR of a given straddling stock in a given year, then this would mean that 20% of the shared stock’s abundance is located on the high seas (*14*). The SSR concept was derived from J. Nash’s “threat point” in game theory, which defines the minimum payoff a player requires to cooperate with other players (*99*, *100*). This framework has been applied to contribute to understanding the challenges posed by climate change for internationally shared stocks (*14*, *48*, *98*). For each stock, we calculated the average SSR during the historical period from 1951 to 2014 ( ${\text{SSR}}_{h}$ ) and two future time periods ( ${\text{SSR}}_{f}$ ): 2021 to 2040, representing the 2030 average, and 2041 to 2060 (representing the 2050 average). We also calculated the historical variability of the stock using the SD (σ) of the annual SSR time series during the historical period ( ${\text{SSR}}_{h}$).

We used these time series to estimate the number of straddling stocks that would shift their distribution in each time period and climate change scenario. We defined a significant change in a straddling SSR as occurring when the projected ${\text{SSR}}_{f}$ fell outside two SDs of the historical mean (95% probability), i.e., a significant stock shift was identified if ${\text{SSR}}_{f}$ > ( ${\text{SSR}}_{h}$ + 2σ) or ${\text{SSR}}_{f}$ < ( ${\text{SSR}}_{h}$ − 2σ). We then categorized the identified stock shifts between EEZs and the high seas into three categories: (i) a gain: an increase in the proportion of the abundance of a given stock was higher in the high seas or EEZs, (ii) a loss: a decrease in the proportion of the abundance of a given stock in the high seas or EEZs, and (iii) no change: the projected SSR $\left({\text{SSR}}_{f}\right)$ was not significantly different from the historical SSR $\left({\text{SSR}}_{h}\right)$ for both the EEZs and the high seas. We applied the same analysis for highly migratory straddling stocks shared between neighboring RFMOs (*21*) to assess shifts between management frameworks.

To estimate the magnitude of shifts, we computed the percentage change in SSR ( $\Delta {\text{SSR}}_{s}$ ) by 2030 and 2050 $\left(\Delta {\text{SSR}}_{f}\right)$ , relative to the average historical time period ( $\Delta {\text{SSR}}_{h}$ ) as follows

$$\Delta {\text{SSR}}_{s}=\frac{{\text{SSR}}_{f}-{\text{SSR}}_{h}}{{\text{SSR}}_{h}}\times 100$$

All projections were conducted using three ESMs, and we used ensemble mean projections of SSR to account for structural uncertainties in climate and ocean projections (*29*). All analyses were run using the statistical software R (*101*) version 4.3.1 (2023-06-16, Beagle Scouts) and associated packages (table S3).
