# Supplementary material for: Climate change drives shifts in straddling fish stocks in the world’s ocean
Source: Sci Adv. 2025 Jul 30;11(31):eadq5976. doi: 10.1126/sciadv.adq5976 (PMC12309684; doi:10.1126/sciadv.adq5976)
Supplement: Supplementary file 1 — Figs. S1 to S8 Tables S1 to S3 [file sciadv.adq5976_sm.pdf]

Science Advances
AAAS

Supplementary Materials for

Climate change drives shifts in straddling fish stocks in the world's ocean

Juliano Palacios-Abrantes *et al.*

Corresponding author: Juliano Palacios-Abrantes, j.palacios@oceans.ubc.ca

Sci. Adv. **11**, eadq5976 (2025)
DOI: 10.1126/sciadv.adq5976

This PDF file includes:

Figs. S1 to S8
Tables S1 to S3

Supplementary Figures

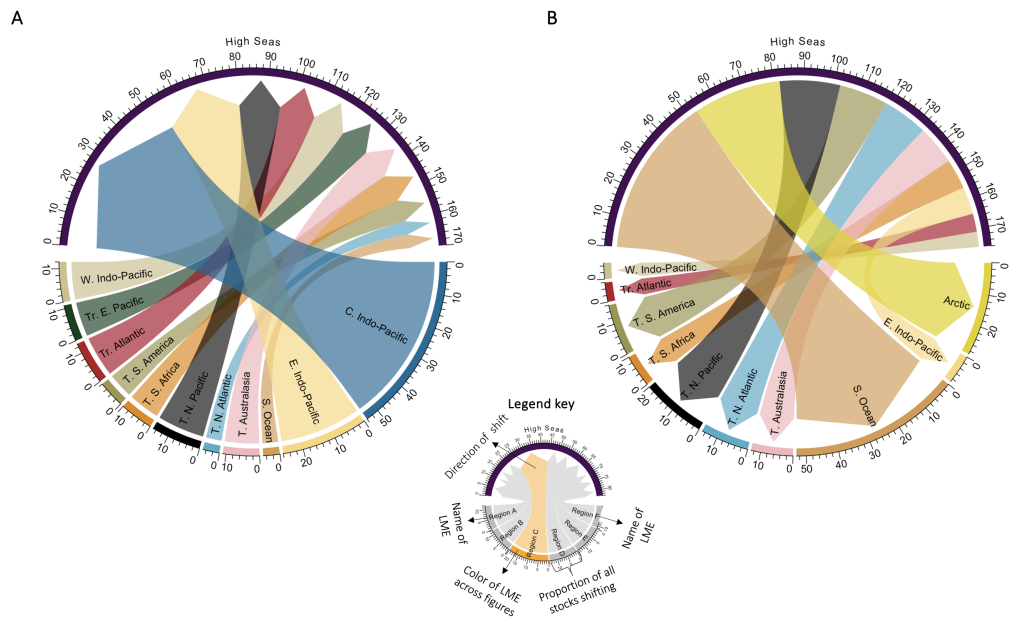

Figure S1: Percentage of total straddling stocks per region projected to undergo significant changes in stock share ratio (SSR) between Exclusive Economic Zones (EEZs) and the high seas by 2030 under the high emissions scenario. A) Arrows indicate stocks shifting from EEZs to the high seas, (B) Arrows indicate stocks shifting from the high seas to EEZs. Values represent the percentage of stocks experiencing significant SSR shifts relative to the total number of straddling stocks in each region. The width of each arrow link width corresponds to the proportion of stocks shifting, with wider links indicating a larger proportion. Arrows are color-coded by region for clarity. Abbreviations: C = Central; E = Eastern; N = North; S = Southern; T = Temperate; Tr = Tropical; W = West. See table S2 for a list of EEZs within each region.

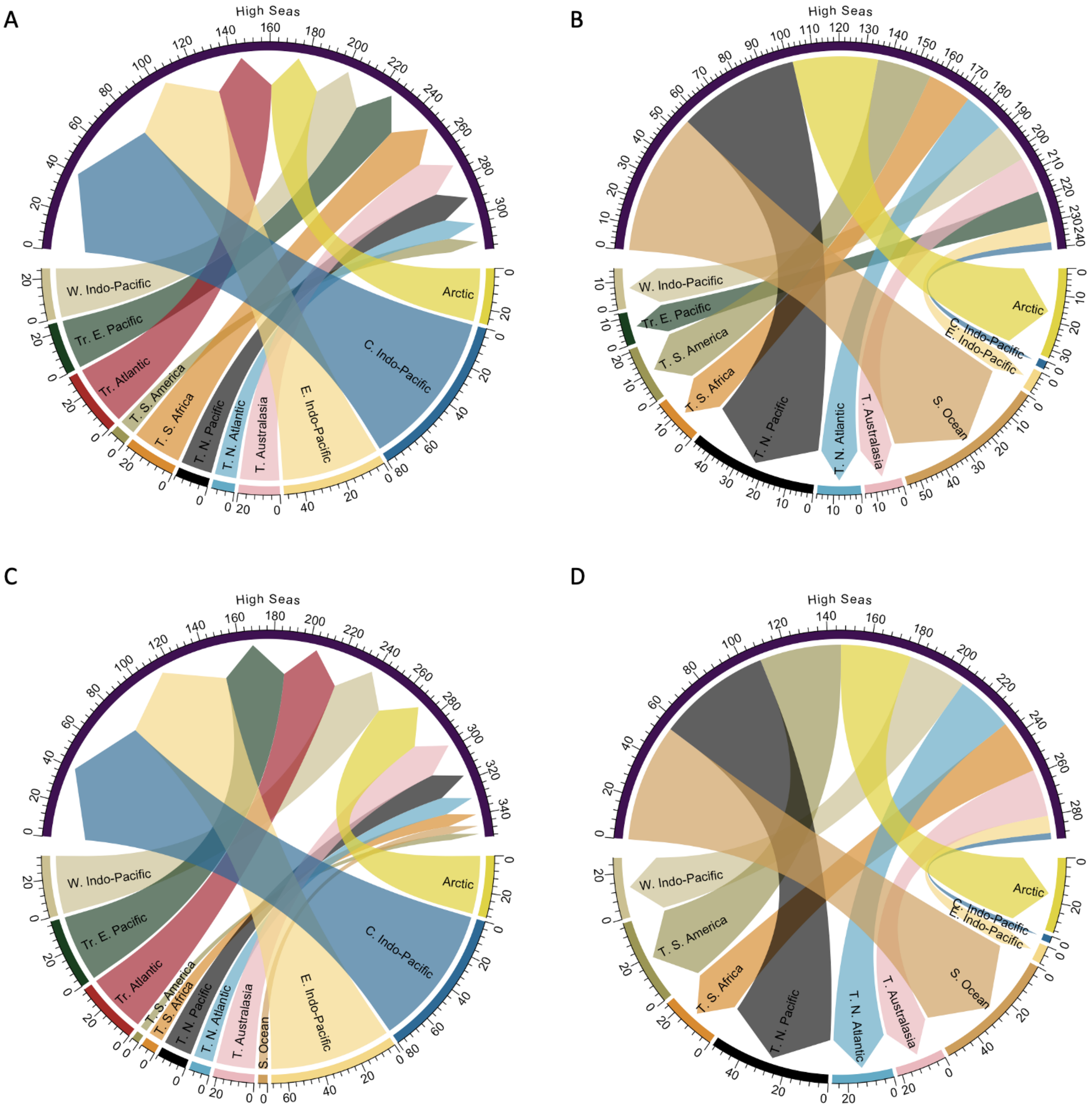

Figure S2: Percentage of total straddling stocks per region projected to undergo significant changesin stock share ratio (SSR) between Exclusive Economic Zones (EEZs) and the high seas by 2050 under both emissions scenarios. A) Shifts from EEZ to the high seas under the low emission scenario, B) shifts from the high seas to EEZ under low emisison scenario, C) shifts from EEZ to the high seas under high emsission scenario, D) shifts from the high seas to EEZ, under high emission scenario. Values represent the percentage of stocks experiencing significant SSR shifts relative to the total number of straddling stocks in each region. Arrows represent directionality and width percentage of stocks shifting to the neighboring zone (EEZ/high seas). The width of each arrow link width corresponds to the proportion of stocks shifting, with wider links indicating a larger proportion. Arrows are color-coded by region for clarity. Abbreviations: C = Central; E = Eastern; N = North; S = Southern; T = Temperate; Tr = Tropical; W = West. See table S2 for a list of EEZs within each region.

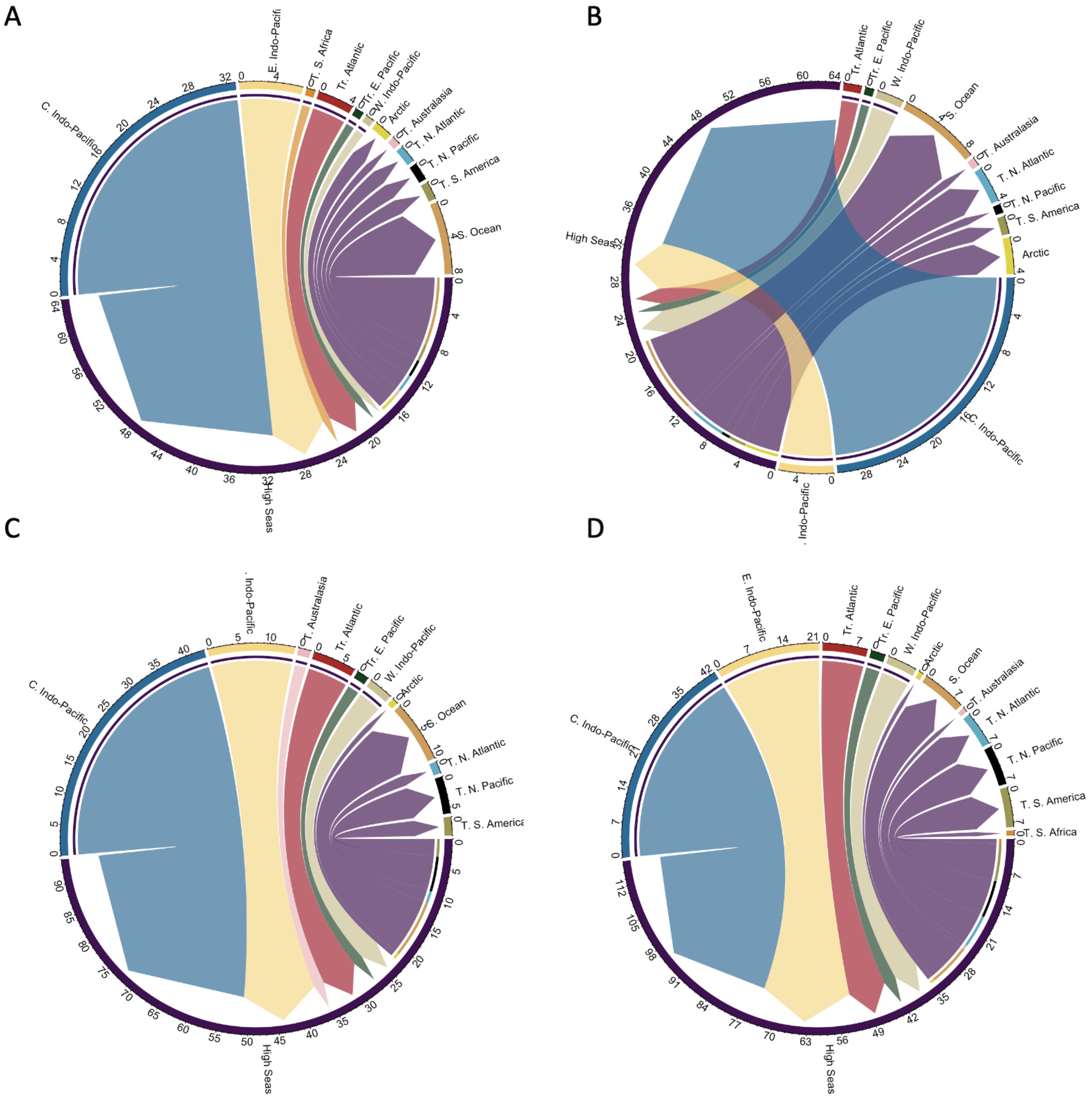

Figure S3: Net changes in Stock Share Ratio (SSR) between Exclusive Economic Zones (EEZ) and the high seas by the middle of the 21st century under both emission scenarios. A) Shifts by 2030 under the low emission scenario, B) Shifts by 2030 under high emsission scenario, C) shifts by 2050 under the low emission scenario, B) shifts by 2050 under high emsission scenario. Links colored by region. Net changes = stock gains - stock losses. Numbers on the axis and size of links represent the percentage of stocks shifting and the arrow shows the direction of the shift.

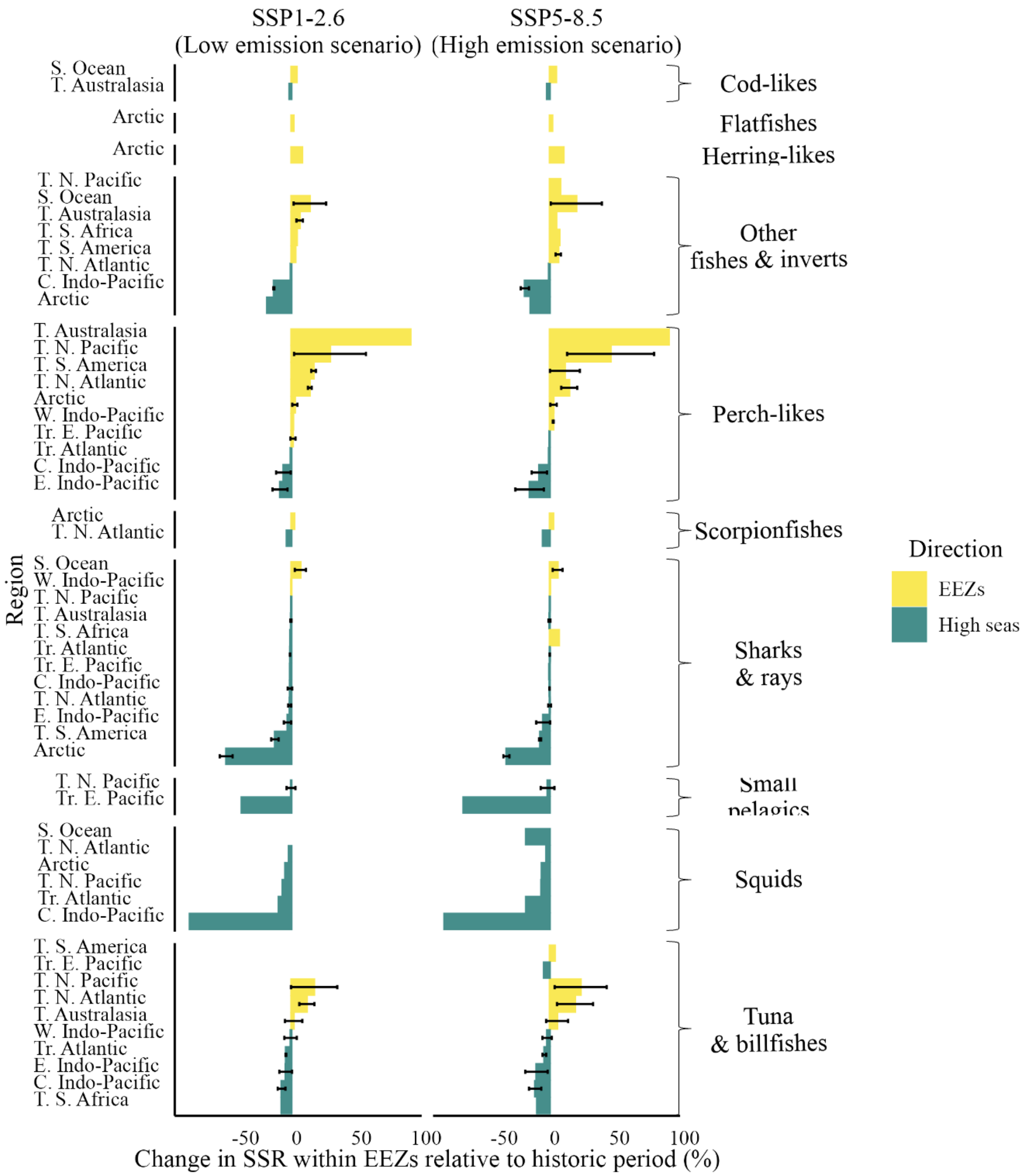

Figure S4: Average percentage change in Stock Share Ratio (SSR) across commercial groups within EEZs by 2050. Positive changes indicate an increase in the proportion of stocks within EEZs (yellow), while negative changes indicate an increase in the proportion of stocks in the high seas (green). Error bars represent the standard deviation of SSR changes across shifting stocks within each commercial group. Only commercial groups with shifting stocks are shown for each time period. Abbreviations: C = Central; E = Eastern; N = North; S = Southern; T = Temperate; Tr = Tropical W = West.

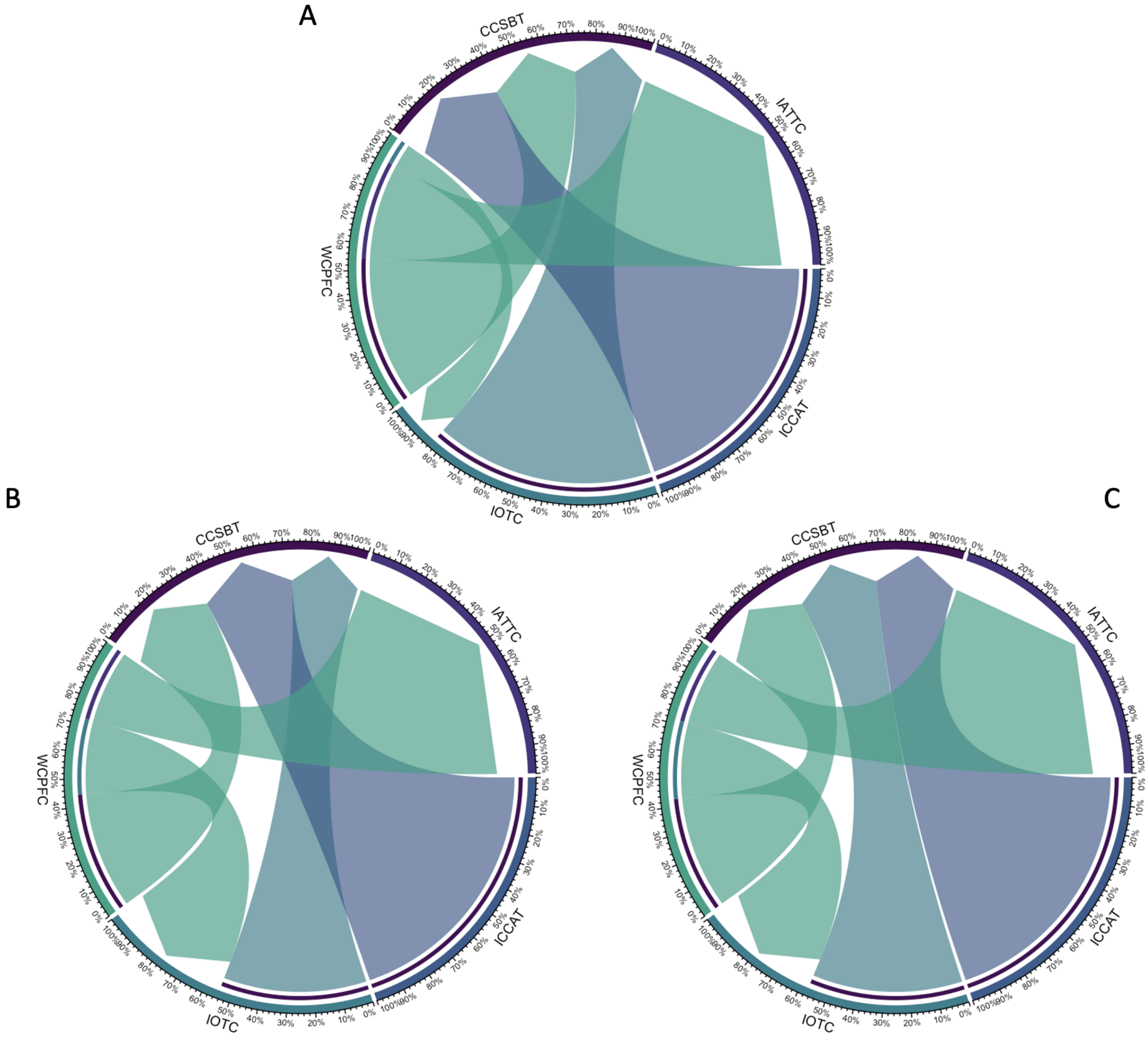

Figure S5: Net changes in Stock Share Ratio (SSR) of highly migratory straddling stocks between neighboring Regional Fisheries Management Organizations (RFMOs). A) By 2030 under the high emission scenario, B) by 2050 under the low emission scenario, C) by 2050 under the high emission scenario. Links colored by RFMO. Net changes = stock gains - stock losses. Numbers on the axis and size of links represent the percentage of stocks shifting and the arrow shows the direction of the shift. The Commission for the Conservation of Southern Bluefin Tuna (CCSBT), The Inter-American Tropical Tuna Commission (IATTC), The International Commission for the Conservation of Atlantic Tunas (ICCAT), The Indian Ocean Tuna Commission (IOTC), and The Western and Central Pacific Fisheries Commission (WCPFC).

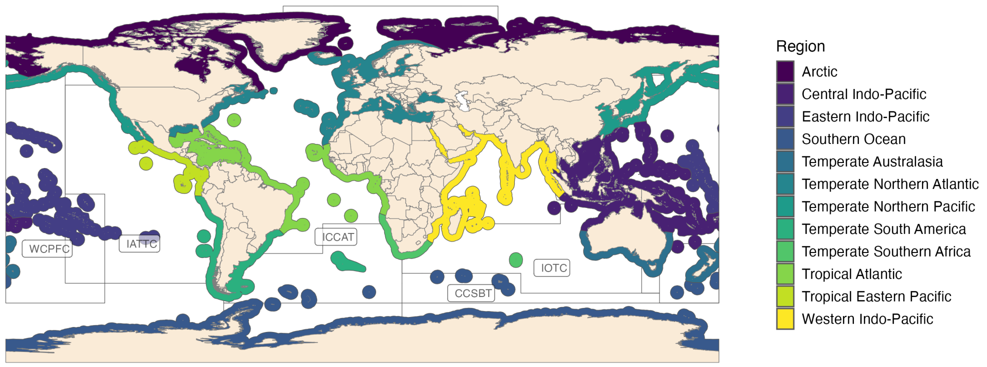

Figure S6: Map showing the five different tuna RFMO and EEZs classifications according to the marine realm established by Spalding et al (88). The Commission for the Conservation of Southern Bluefin Tuna (CCSBT), The Inter-American Tropical Tuna Commission (IATTC), The International Commission for the Conservation of Atlantic Tunas (ICCAT), The Indian Ocean Tuna Commission (IOTC), and The Western and Central Pacific Fisheries Commission (WCPFC). Note that CCSBT has no defined convention area, thus, for this analysis, CCSBT area has been considered the areas south of IATTC, ICCAT, IOTC and WCPFC convention areas' southern limits.

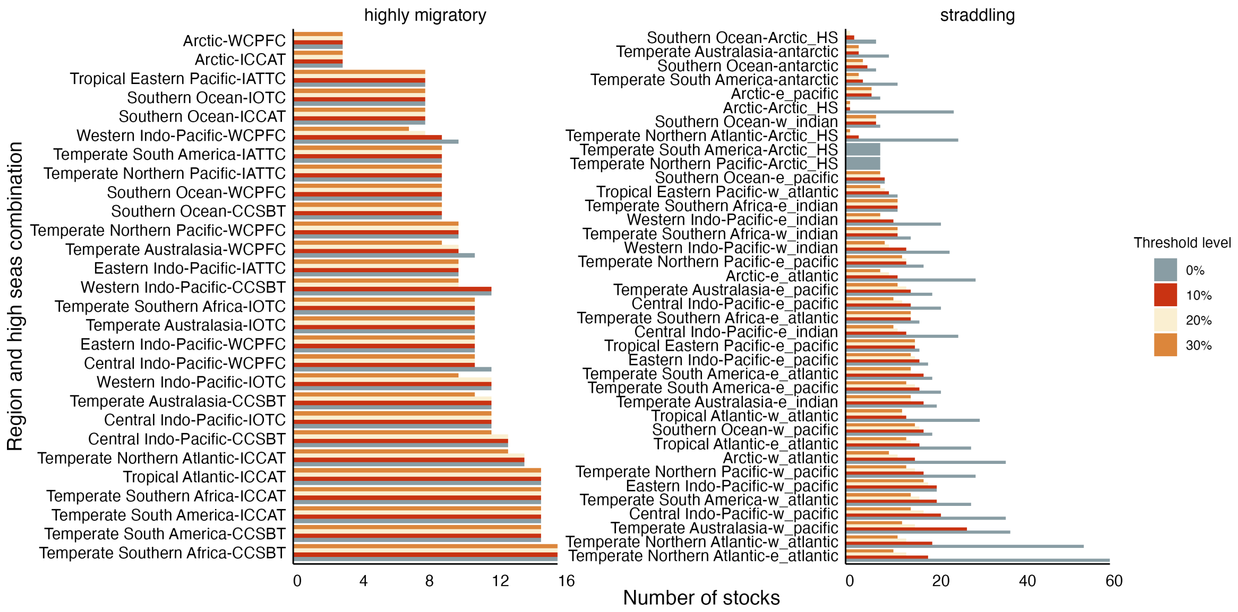

Figure S7: Sensitivity analysis for spatial threshold. Each level represents a minimum percentage of occurrence between Region and the high seas. For example, the 10% threshold means that both the Region and the the high seas have at least 10% of the shared stock. For this study we used the 10% (red) threshold, the 0% (gray) represents no threshold. Analysis done with GFDL under low emission scenario at the early time period (see methods)

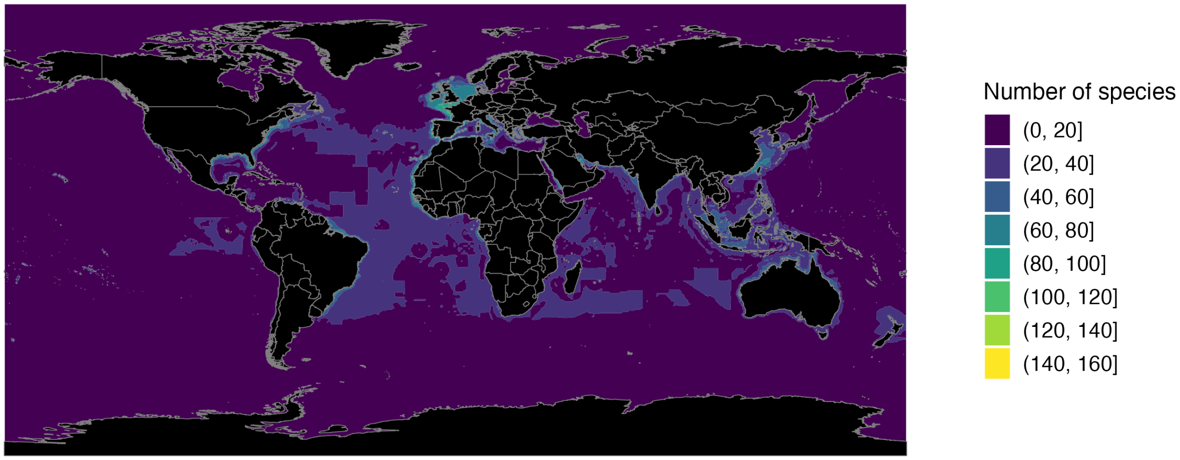

Figure S8: Map showing the number of transboundary species (n = 633) present in each cell of the world. Gridcell scale 0.5 latitude by 0.5 longitude. The original 633 species from Palacios-Abrantes et al (4) were then used to estimate the number of straddling stocks.

Supplementary Tables

Table S1: List of straddling species identified in the present study grouped by “commercial group” based on the Sea Around Us classification.

Scientific name	Common name	Commercial group
Acanthocybium solandri	Wahoo	Perch-likes
Alepocephalus bairdii	Bairds slickhead	Salmon, smelts, etc
Alopias vulpinus	Thresher	Sharks & rays
Alosa pseudoharengus	Alewife	Herring-likes
Auxis thazard	Frigate tuna	Tuna & billfishes
Boreogadus saida	Polar cod	Cod-likes
Brama brama	Atlantic pomfret	Perch-likes
Carcharhinus falciformis	Silky shark	Sharks & rays
Carcharhinus longimanus	Oceanic whitetip shark	Sharks & rays
Carcharhinus plumbeus	Sandbar shark	Sharks & rays
Carcharodon carcharias	Great white shark	Sharks & rays
Centrophorus squamosus	Leafscale gulper shark	Sharks & rays
Centroscymnus coelolepis	Portuguese dogfish	Sharks & rays
Centroscymnus crepidater	Longnose velvet dogfish	Sharks & rays
Cetorhinus maximus	Basking shark	Sharks & rays
Cololabis saira	Pacific saury	Small pelagics
Coryphaena hippurus	Common dolphinfish	Perch-likes
Dalatias licha	Kitefin shark	Sharks & rays
Dissostichus eleginoides	Patagonian toothfish	Perch-likes
Epigonus telescopus	Black cardinal fish	Perch-likes
Euthynnus affinis	Kawakawa	Tuna & billfishes
Galeorhinus galeus	Tope shark	Sharks & rays
Genypterus blacodes	Pink cusk-eel	Other fishes & inverts
Hippoglossoides platessoides	American plaice	Flatfishes
Hoplostethus atlanticus	Orange roughy	Other fishes & inverts
Hoplostethus mediterraneus mediterraneus	Mediterranean slimehead	Other fishes & inverts
Illex illecebrosus	Northern shortfin squid	Squids
Istiompax indica	Black marlin	Tuna & billfishes
Istiophorus platypterus	Indo-Pacific sailfish	Tuna & billfishes
Isurus oxyrinchus	Shortfin mako	Sharks & rays
Isurus paucus	Longfin mako	Sharks & rays
Kajikia albida	Atlantic white marlin	Tuna & billfishes
Kajikia audax	Striped marlin	Tuna & billfishes
Katsuwonus pelamis	Skipjack tuna	Tuna & billfishes
Lamna nasus	Porbeagle	Sharks & rays
Lepidocybium flavobrunneum	Escolar	Perch-likes
Lobotes surinamensis	Tripletail	Perch-likes
Makaira nigricans	Blue marlin	Tuna & billfishes
Mallotus villosus	Capelin	Salmon, smelts, etc
Martialia hyadesi	Sevenstar flying squid	Squids
Merluccius australis	Southern hake	Cod-likes
Micromesistius australis	Southern blue whiting	Cod-likes
Mola mola	Ocean sunfish	Other fishes & inverts
Odontesthes regia	Chilean silverside	Small pelagics

Paralabrax humeralis	Peruvian rock seabass	Perch-likes
Prionace glauca	Blue shark	Sharks & rays
Regalecus glesne	King of herrings	Other fishes & inverts
Ruvettus pretiosus	Oilfish	Perch-likes
Sarda sarda	Atlantic bonito	Tuna & billfishes
Scomber japonicus	Chub mackerel	Perch-likes
Scomber scombrus	Atlantic mackerel	Perch-likes
Sebastes mentella	Beaked redfish	Scorpionfishes
Sebastes norvegicus	Golden redfish	Scorpionfishes
Sphyrna zygaena	Smooth hammerhead	Sharks & rays
Squalus acanthias	Piked dogfish	Sharks & rays
Tetrapturus angustirostris	Shortbill spearfish	Tuna & billfishes
Thunnus alalunga	Albacore	Tuna & billfishes
Thunnus albacares	Yellowfin tuna	Tuna & billfishes
Thunnus maccoyii	Southern bluefin tuna	Tuna & billfishes
Thunnus obesus	Bigeye tuna	Tuna & billfishes
Thunnus orientalis	Pacific bluefin tuna	Tuna & billfishes
Thunnus thynnus	Atlantic bluefin tuna	Tuna & billfishes
Todarodes pacificus	Japanese flying squid	Squids
Todarodes sagittatus	European flying squid	Squids
Trachurus murphyi	Chilean jack mackerel	Perch-likes
Trachurus trachurus	Atlantic horse mackerel	Perch-likes
Xiphias gladius	Swordfish	Tuna & billfishes

The global EEZ shapefile (updated 1 July 2015) and the RFMOs shapefiles were provided by SAU (Available from https ://www.seaaroundus.org). It is important to note that the data subdivides the EEZs of 198 coastal states into 280 regions (e.g., Mexico's EEZ is divided in Mexico Pacificand Mexico Atlantic), including islands territories. Finally, the MEOW shapefile according to Spalding et al (88) was provided by World Widlife Fund and The Nature Conservancy (Available from https://www.worldwildlife.org/publications/marine-ecoregions-of-the-world-a-bioregionalization-of-coastal-and-shelf-areas).

Table S2: List of Exclusive Economic Zones (EEZs) according to the Sea Around Us (www.searoundus.org) and their Realm according to Spalding et al (88).

Realm	Exclusive Economic Zone
Arctic	Canada (Arctic)
Arctic	Canada (East Coast)
Arctic	Greenland
Arctic	Iceland
Arctic	Jan Mayen Isl. (Norway)
Arctic	Norway
Arctic	Russia (Barents Sea)
Arctic	Russia (Far East)
Arctic	Russia (Kara Sea)
Arctic	Russia (Laptev to Chukchi Sea)
Arctic	Svalbard Isl. (Norway)
Arctic	USA (Alaska, Arctic)
Arctic	USA (Alaska, Subarctic)
Central Indo-Pacific	American Samoa
Central Indo-Pacific	Australia

Central Indo-Pacific	Brunei Darussalam
Central Indo-Pacific	Cambodia
Central Indo-Pacific	China
Central Indo-Pacific	Christmas Isl. (Australia)
Central Indo-Pacific	Cocos (Keeling) Isl. (Australia)
Central Indo-Pacific	Cook Islands
Central Indo-Pacific	Fiji
Central Indo-Pacific	Guam (USA)
Central Indo-Pacific	Hong Kong (China)
Central Indo-Pacific	Indonesia (Central)
Central Indo-Pacific	Indonesia (Eastern)
Central Indo-Pacific	Indonesia (Indian Ocean)
Central Indo-Pacific	Japan (Daito Islands)
Central Indo-Pacific	Japan (Ogasawara Islands)
Central Indo-Pacific	Japan (main islands)
Central Indo-Pacific	Lord Howe Isl. (Australia)
Central Indo-Pacific	Malaysia (Peninsula East)
Central Indo-Pacific	Malaysia (Peninsula West)
Central Indo-Pacific	Malaysia (Sabah)
Central Indo-Pacific	Malaysia (Sarawak)
Central Indo-Pacific	Marshall Isl.
Central Indo-Pacific	Micronesia (Federated States of)
Central Indo-Pacific	New Caledonia (France)
Central Indo-Pacific	New Zealand
Central Indo-Pacific	Niue (New Zealand)
Central Indo-Pacific	Norfolk Isl. (Australia)
Central Indo-Pacific	Northern Marianas (USA)
Central Indo-Pacific	Palau
Central Indo-Pacific	Papua New Guinea
Central Indo-Pacific	Philippines
Central Indo-Pacific	Singapore
Central Indo-Pacific	Solomon Isl.
Central Indo-Pacific	Taiwan
Central Indo-Pacific	Thailand (Andaman Sea)
Central Indo-Pacific	Thailand (Gulf of Thailand)
Central Indo-Pacific	Timor Leste
Central Indo-Pacific	Tonga
Central Indo-Pacific	Vanuatu
Central Indo-Pacific	Viet Nam
Central Indo-Pacific	Wallis & Futuna Isl. (France)
Eastern Indo-Pacific	American Samoa
Eastern Indo-Pacific	Cook Islands
Eastern Indo-Pacific	Easter Isl. (Chile)
Eastern Indo-Pacific	Fiji
Eastern Indo-Pacific	French Polynesia
Eastern Indo-Pacific	Hawaii Main Islands (USA)
Eastern Indo-Pacific	Hawaii Northwest Islands (USA)
Eastern Indo-Pacific	Howland & Baker Isl. (USA)
Eastern Indo-Pacific	Jarvis Isl. (USA)
Eastern Indo-Pacific	Johnston Atoll (USA)

Eastern Indo-Pacific	Kiribati (Gilbert Islands)
Eastern Indo-Pacific	Kiribati (Line Islands)
Eastern Indo-Pacific	Kiribati (Phoenix Islands)
Eastern Indo-Pacific	Marshall Isl.
Eastern Indo-Pacific	Micronesia (Federated States of)
Eastern Indo-Pacific	Nauru
Eastern Indo-Pacific	Niue (New Zealand)
Eastern Indo-Pacific	Palmyra Atoll & Kingman Reef (USA)
Eastern Indo-Pacific	Pitcairn (UK)
Eastern Indo-Pacific	Samoa
Eastern Indo-Pacific	Solomon Isl.
Eastern Indo-Pacific	Tokelau (New Zealand)
Eastern Indo-Pacific	Tonga
Eastern Indo-Pacific	Tuvalu
Eastern Indo-Pacific	Wake Isl. (USA)
Eastern Indo-Pacific	Wallis & Futuna Isl. (France)
Southern Ocean	Bouvet Isl. (Norway)
Southern Ocean	Crozet Isl. (France)
Southern Ocean	Heard & McDonald Isl. (Australia)
Southern Ocean	Kerguelen Isl. (France)
Southern Ocean	Macquarie Isl. (Australia)
Southern Ocean	New Zealand
Southern Ocean	Prince Edward Isl. (South Africa)
Southern Ocean	South Georgia & Sandwich Isl. (UK)
Southern Ocean	South Orkney Islands (UK)
Temperate Australasia	Australia
Temperate Australasia	Kermadec Isl. (New Zealand)
Temperate Australasia	Lord Howe Isl. (Australia)
Temperate Australasia	New Zealand
Temperate Australasia	Norfolk Isl. (Australia)
Temperate Northern Atlantic	Albania
Temperate Northern Atlantic	Algeria
Temperate Northern Atlantic	Azores Isl. (Portugal)
Temperate Northern Atlantic	Bahamas
Temperate Northern Atlantic	Balearic Island (Spain)
Temperate Northern Atlantic	Belgium
Temperate Northern Atlantic	Bosnia & Herzegovina
Temperate Northern Atlantic	Bulgaria
Temperate Northern Atlantic	Canada (East Coast)
Temperate Northern Atlantic	Canary Isl. (Spain)
Temperate Northern Atlantic	Channel Isl. (UK)
Temperate Northern Atlantic	Corsica (France)
Temperate Northern Atlantic	Crete (Greece)
Temperate Northern Atlantic	Croatia
Temperate Northern Atlantic	Cyprus (North)
Temperate Northern Atlantic	Cyprus (South)
Temperate Northern Atlantic	Denmark (Baltic Sea)
Temperate Northern Atlantic	Denmark (North Sea)
Temperate Northern Atlantic	Egypt (Mediterranean)
Temperate Northern Atlantic	Estonia

Temperate Northern Atlantic	Faeroe Isl. (Denmark)
Temperate Northern Atlantic	Finland
Temperate Northern Atlantic	France (Atlantic Coast)
Temperate Northern Atlantic	France (Mediterranean)
Temperate Northern Atlantic	Gaza Strip
Temperate Northern Atlantic	Georgia
Temperate Northern Atlantic	Germany (Baltic Sea)
Temperate Northern Atlantic	Germany (North Sea)
Temperate Northern Atlantic	Greece
Temperate Northern Atlantic	Iceland
Temperate Northern Atlantic	Ireland
Temperate Northern Atlantic	Israel (Mediterranean)
Temperate Northern Atlantic	Italy
Temperate Northern Atlantic	Latvia
Temperate Northern Atlantic	Lebanon
Temperate Northern Atlantic	Libya
Temperate Northern Atlantic	Lithuania
Temperate Northern Atlantic	Madeira Isl. (Portugal)
Temperate Northern Atlantic	Malta
Temperate Northern Atlantic	Mauritania
Temperate Northern Atlantic	Mexico (Atlantic)
Temperate Northern Atlantic	Montenegro
Temperate Northern Atlantic	Morocco (Central)
Temperate Northern Atlantic	Morocco (Mediterranean)
Temperate Northern Atlantic	Morocco (South)
Temperate Northern Atlantic	Netherlands
Temperate Northern Atlantic	Norway
Temperate Northern Atlantic	Poland
Temperate Northern Atlantic	Portugal
Temperate Northern Atlantic	Romania
Temperate Northern Atlantic	Russia (Baltic Sea)
Temperate Northern Atlantic	Russia (Barents Sea)
Temperate Northern Atlantic	Russia (Black Sea)
Temperate Northern Atlantic	Saint Pierre & Miquelon (France)
Temperate Northern Atlantic	Sardinia (Italy)
Temperate Northern Atlantic	Sicily (Italy)
Temperate Northern Atlantic	Slovenia
Temperate Northern Atlantic	Spain (Mediterranean and Gulf of Cadiz)
Temperate Northern Atlantic	Spain (Northwest)
Temperate Northern Atlantic	Sweden (Baltic)
Temperate Northern Atlantic	Sweden (West Coast)
Temperate Northern Atlantic	Syria
Temperate Northern Atlantic	Tunisia
Temperate Northern Atlantic	Turkey (Black Sea)
Temperate Northern Atlantic	Turkey (Marmara Sea)
Temperate Northern Atlantic	Turkey (Mediterranean Sea)
Temperate Northern Atlantic	USA (East Coast)
Temperate Northern Atlantic	USA (Gulf of Mexico)
Temperate Northern Atlantic	Ukraine
Temperate Northern Atlantic	United Kingdom

Temperate Northern Pacific	Canada (Pacific)
Temperate Northern Pacific	China
Temperate Northern Pacific	Japan (main islands)
Temperate Northern Pacific	Korea (North)
Temperate Northern Pacific	Korea (South)
Temperate Northern Pacific	Mexico (Pacific)
Temperate Northern Pacific	Russia (Far East)
Temperate Northern Pacific	Taiwan
Temperate Northern Pacific	USA (Alaska, Subarctic)
Temperate Northern Pacific	USA (West Coast)
Temperate South America	Argentina
Temperate South America	Brazil
Temperate South America	Chile
Temperate South America	Desventuradas Isl. (Chile)
Temperate South America	Falkland Isl. (UK)
Temperate South America	Juan Fernandez Islands (Chile)
Temperate South America	Peru
Temperate South America	Tristan da Cunha Isl. (UK)
Temperate South America	Uruguay
Temperate Southern Africa	Angola
Temperate Southern Africa	Namibia
Temperate Southern Africa	South Africa (Atlantic Coast)
Temperate Southern Africa	South Africa (Indian Ocean Coast)
Temperate Southern Africa	St Paul & Amsterdam Isl. (France)
Tropical Atlantic	Angola
Tropical Atlantic	Anguilla (UK)
Tropical Atlantic	Antigua & Barbuda
Tropical Atlantic	Aruba (Netherlands)
Tropical Atlantic	Ascension Isl. (UK)
Tropical Atlantic	Bahamas
Tropical Atlantic	Barbados
Tropical Atlantic	Belize
Tropical Atlantic	Benin
Tropical Atlantic	Bermuda (UK)
Tropical Atlantic	Bonaire (Netherlands)
Tropical Atlantic	Brazil
Tropical Atlantic	Brazil (Fernando de Noronha)
Tropical Atlantic	Brazil (St Paul and St. Peter Archipelago)
Tropical Atlantic	British Virgin Isl. (UK)
Tropical Atlantic	Cameroon
Tropical Atlantic	Cape Verde
Tropical Atlantic	Cayman Isl. (UK)
Tropical Atlantic	Colombia (Caribbean)
Tropical Atlantic	Congo (ex-Zaire)
Tropical Atlantic	Congo, R. of
Tropical Atlantic	Costa Rica (Caribbean)
Tropical Atlantic	Cuba
Tropical Atlantic	Curacao (Netherlands)
Tropical Atlantic	Côte d'Ivoire
Tropical Atlantic	Dominica

Tropical Atlantic	Dominican Republic
Tropical Atlantic	Equatorial Guinea
Tropical Atlantic	French Guiana
Tropical Atlantic	Gabon
Tropical Atlantic	Gambia
Tropical Atlantic	Ghana
Tropical Atlantic	Grenada
Tropical Atlantic	Guadeloupe (France)
Tropical Atlantic	Guatemala (Caribbean)
Tropical Atlantic	Guinea
Tropical Atlantic	Guinea-Bissau
Tropical Atlantic	Guyana
Tropical Atlantic	Haiti
Tropical Atlantic	Honduras (Caribbean)
Tropical Atlantic	Jamaica
Tropical Atlantic	Liberia
Tropical Atlantic	Martinique (France)
Tropical Atlantic	Mauritania
Tropical Atlantic	Mexico (Atlantic)
Tropical Atlantic	Montserrat (UK)
Tropical Atlantic	Morocco (South)
Tropical Atlantic	Nicaragua (Caribbean)
Tropical Atlantic	Nigeria
Tropical Atlantic	Panama (Caribbean)
Tropical Atlantic	Panama (Pacific)
Tropical Atlantic	Puerto Rico (USA)
Tropical Atlantic	Saba and Sint Eustaius (Netherlands)
Tropical Atlantic	Saint Helena (UK)
Tropical Atlantic	Saint Kitts & Nevis
Tropical Atlantic	Saint Lucia
Tropical Atlantic	Saint Vincent & the Grenadines
Tropical Atlantic	Sao Tome & Principe
Tropical Atlantic	Senegal
Tropical Atlantic	Sierra Leone
Tropical Atlantic	Sint Maarten (Netherlands)
Tropical Atlantic	St Barthelemy (France)
Tropical Atlantic	St Martin (France)
Tropical Atlantic	Suriname
Tropical Atlantic	Togo
Tropical Atlantic	Trindade & Martim Vaz Isl. (Brazil)
Tropical Atlantic	Trinidad & Tobago
Tropical Atlantic	Turks & Caicos Isl. (UK)
Tropical Atlantic	US Virgin Isl.
Tropical Atlantic	USA (East Coast)
Tropical Atlantic	USA (Gulf of Mexico)
Tropical Atlantic	Venezuela
Tropical Eastern Pacific	Clipperton Isl. (France)
Tropical Eastern Pacific	Colombia (Pacific)
Tropical Eastern Pacific	Costa Rica (Pacific)
Tropical Eastern Pacific	Ecuador

Tropical Eastern Pacific	El Salvador
Tropical Eastern Pacific	Galapagos Isl. (Ecuador)
Tropical Eastern Pacific	Guatemala (Pacific)
Tropical Eastern Pacific	Honduras (Pacific)
Tropical Eastern Pacific	Mexico (Pacific)
Tropical Eastern Pacific	Nicaragua (Pacific)
Tropical Eastern Pacific	Panama (Pacific)
Tropical Eastern Pacific	Peru
Western Indo-Pacific	Andaman & Nicobar Isl. (India)
Western Indo-Pacific	Bahrain
Western Indo-Pacific	Bangladesh
Western Indo-Pacific	Chagos Archipelago (UK)
Western Indo-Pacific	Comoros Isl.
Western Indo-Pacific	Djibouti
Western Indo-Pacific	Egypt (Red Sea)
Western Indo-Pacific	Eritrea
Western Indo-Pacific	Glorieuse Islands (France)
Western Indo-Pacific	India (mainland)
Western Indo-Pacific	Indonesia (Indian Ocean)
Western Indo-Pacific	Iran (Persian Gulf)
Western Indo-Pacific	Iran (Sea of Oman)
Western Indo-Pacific	Iraq
Western Indo-Pacific	Israel (Red Sea)
Western Indo-Pacific	Jordan
Western Indo-Pacific	Kenya
Western Indo-Pacific	Kuwait
Western Indo-Pacific	Madagascar
Western Indo-Pacific	Malaysia (Peninsula West)
Western Indo-Pacific	Maldives
Western Indo-Pacific	Mauritius
Western Indo-Pacific	Mayotte (France)
Western Indo-Pacific	Mozambique
Western Indo-Pacific	Mozambique Channel Isl. (France)
Western Indo-Pacific	Myanmar
Western Indo-Pacific	Oman
Western Indo-Pacific	Oman (Musandam)
Western Indo-Pacific	Pakistan
Western Indo-Pacific	Qatar
Western Indo-Pacific	Réunion (France)
Western Indo-Pacific	Saudi Arabia (Persian Gulf)
Western Indo-Pacific	Saudi Arabia (Red Sea)
Western Indo-Pacific	Seychelles
Western Indo-Pacific	Somalia
Western Indo-Pacific	South Africa (Indian Ocean Coast)
Western Indo-Pacific	Sri Lanka
Western Indo-Pacific	Sudan
Western Indo-Pacific	Tanzania
Western Indo-Pacific	Thailand (Andaman Sea)
Western Indo-Pacific	Tromelin Isl. (France)
Western Indo-Pacific	United Arab Emirates

Western Indo-Pacific	United Arab Emirates (Fujairah)
Western Indo-Pacific	Yemen (Arabian Sea)
Western Indo-Pacific	Yemen (Red Sea)

Table S3: List of R packages used in the current study.

Package	Source
readxl	Wickham, H. and Bryan, J., 2022. Package readxl; Read Excel Files. CRAN.
data.table	Dowle, M. and Srinivasan, A., 2022. Package data.table; Extension of "data.frame." CRAN.
wesanderson	Ram, K., Wickham, H., Richards, C. and Baggett, A., 2018. Package wesanderson; A Wes Anderson Palette Generator. CRAN.
tidyverse	Wickham, H., 2022. Pakcage tidyverse; Easily Install and Load the "Tidyverse." CRAN.
janitor	Firke, S., Haid, C., Knight, R. and Denney, B., 2018. Package janitor; Simple tools for examining and cleaning dirty data. CRAN.
tidytext	Queiroz, G. D., Hvitfeldt, E., Keyes, O., Misra, K., Mastny, T., Erickson, J., Robinson, D. and Silge, J., 2019. Package tidytext; Text Mining using "dplyr", "ggplot2", and Other Tidy Tools. CRAN.
ggrepel	Slowikowski, K., 2020. Package ggrepel: Automatically Position Non-Overlapping Text Labels with "ggplot2." CRAN.
sf	Pebesma, E., 2022. Package sf; Simple Features for R. CRAN.
sp	Pebesma, E. and Bivand, R., 2023. Package sp; Classes and methods for Spatial Data. CRAN.
doParallel	Daniel, F. and Weston, S., 2022. doParallel: Foreach Parallel Adaptor for the "parallel" Package. CRAN.
rfishbase	Boettiger, C., Chamberlain, S., Lang, D. T. and Wainwright, P., 2019. Package rfishbase; R Interface to "FishBase". CRAN.
zoo	Zeileis, A., Grothendieck, G., Ryan, J. A., Ulrich, J. M. and Andrews, F., 2019. Package zoo; S3 Infrastructure for Regular and Irregular Time Series (Z's Ordered Observations). CRAN.
rnaturalearth	Massicott, P. and South, A., 2023. Package rnaturalearth; World Map Data from Natural Earth. CRAN.
R.matlab	Bengtsson, H., Jacobson, A. and Riedy, J., 2018. Package R.matlab: Read and Write MAT Files and Call MATLAB from Within R. CRAN.
viridis	Garnier, S., 2018. Package viridis; Colorblind-Friendly Color Maps for R. CRAN.
circlize	circlize
